# Different tenogenic differentiation capacities of different mesenchymal stem cells in the presence of BMP-12

**DOI:** 10.1186/s12967-015-0560-7

**Published:** 2015-06-24

**Authors:** Linghui Dai, Xiaoqing Hu, Xin Zhang, Jingxian Zhu, Jiying Zhang, Xin Fu, Xiaoning Duan, Yingfang Ao, Chunyan Zhou

**Affiliations:** Department of Biochemistry and Molecular Biology, Peking University School of Basic Medical Sciences, No. 38 Xueyuan Road, Haidian District, Beijing, 100191 People’s Republic of China; Beijing Key Laboratory of Sports Injuries, Institute of Sports Medicine, Peking University Third Hospital, No. 49 North Garden Road, Haidian District, Beijing, People’s Republic of China

## Abstract

**Background:**

Mesenchymal stem cells (MSCs) are regarded as a promising cell-based therapeutic tool for tendon repair. This study aimed to compare the different tenogenic differentiation capacities of the three types of MSCs in the presence of bone morphogenic protein 12 (BMP-12).

**Methods:**

MSCs were isolated from rat bone marrow (BM), inguinal adipose tissue (AD), and synovium (SM) from the knee joint. MSCs were characterized by morphology, proliferation, trilineage differentiation, and surface marker analysis. Tenogenic differentiation potential was initially assessed using real-time polymerase chain reaction, Western blot, and enzyme-linked immunosorbent assay in vitro. Histological assessments were also performed after subcutaneous implantation of BMP-12 recombinant adenovirus-infected MSCs in nude mice in vivo.

**Results:**

The three types of MSCs exhibited similar fibroblast-like morphology and surface markers but different differentiation potentials toward adipogenic, osteogenic, and chondrogenic lineage fates. Bone marrow-derived MSCs (BM-MSCs) showed the most superior in vitro tenogenic differentiation capacity, followed by synovial membrane-derived MSCs (SM-MSCs) and then adipose-derived MSCs (AD-MSCs). After implantation, all three types of MSC masses infected with BMP-12 recombinant adenovirus emerged in the form of fiber-like matrix, especially in 6-week specimens, compared with the control MSCs in vivo. BM-MSCs and SM-MSCs revealed more intense staining for collagen type I (Col I) compared with AD-MSCs. Differences were not observed between BM-MSCs and SM-MSCs. However, SM-MSCs demonstrated higher proliferation capacity than BM-MSCs.

**Conclusion:**

BM-MSCs exhibited the most superior tenogenic differentiation capacity, followed by SM-MSCs. By contrast, AD-MSCs demonstrated the inferior capacity among the three types of MSCs in the presence of BMP-12 both in vivo and in vitro.

## Background

Tendon injuries are common diseases in the musculoskeletal system. At least 300,000 people undergo surgical tendon repairs each year in the United States [1]. Unfortunately, healing of the injured tendon is poor because of its limited regenerative potential [2]. Moreover, this injury has a long recovery time, ranging from months to years. Healed tendons do not regain their initial properties, and significant dysfunction may ensue [3, 4]. Thus, improving the efficiency of tendon repair is imperative.

Several cytokines, including bone morphogenetic proteins (BMPs), transforming growth factor-beta (TGF-β), insulin-like growth factor (IGF), vascular endothelial growth factor (VEGF), and fibroblast growth factor (FGF) [5–9], can enhance tendon repair. However, not all aforementioned cytokines can promote tenogenic differentiation in cell-based therapeutic methods. Among these cytokines, bone morphogenic protein (BMP-12), also called growth and differentiation factor 7, has shown the most superior capacity to promote tendon repair and tendon-like tissue formation both in vivo and in vitro [8, 10–12]. BMP-12 can also promote tenogenic differentiation of mesenchymal stem cells (MSCs) and even muscle cells [13]. Overall, BMP-12 has remarkable therapeutic potential for tendon repair.

MSCs are also regarded as a promising cell-based therapeutic tool for tendon repair [14–16]. These cells can rapidly proliferate in vitro and can easily be isolated from various tissues, including bone marrow aspirates [17], adipose tissues [18], muscles [19], and synovium [20]. Moreover, MSCs are multipotent and can thus differentiate into several tissues, including bone, cartilage, adipose, and other tissues, under appropriate culture conditions. The capacity of MSCs to differentiate into tenocytes and form tendon tissue has been demonstrated [12, 21–23]. However, MSCs from different tissues are different in terms of proliferation, isolation, and especially differentiation capacity.

An assessment to determine the type of MSCs that exhibits the most superior differentiation capacity toward tenocyte should be conducted to screen the optimum cell source for tenogenic differentiation. Therefore, in this study, we characterized the tenogenic differentiation capacities of rat bone marrow-derived MSCs (BM-MSCs), adipose tissue-derived MSCs (AD-MSCs), and synovial membrane-derived MSCs (SM-MSCs) infected with BMP-12 recombinant adenovirus (Ad-BMP-12) in vitro. We also tested whether the tenocyte-like phenotype is sustained following implantation in nude mice in vivo.

## Methods

### Isolation and culture of MSCs

All MSCs were isolated from Sprague–Dawley rats (100–120 g, n = 5) in this experiment. BM-MSCs were collected from the bone marrow by flushing the femur and tibia with medium, and single-cell suspensions were prepared by repetitively pipetting BM-MSCs through 18-gauge needles as described [20]. After centrifugation, cell pellets were suspended in the growth medium. AD-MSCs were isolated from the inguinal adipose tissue of the rats as previously described [24]. The tissue was minced and digested in phosphate-buffered saline (PBS) containing 0.1% type I collagenase (Sigma-Aldrich, St. Louis, Mo, USA) for 60 min at 37°C with vigorous shaking. After centrifugation, the top lipid layers were removed, and the cells were suspended in the growth medium. SM-MSCs were isolated from the synovium tissue, which comes from the inner side of the medial joint capsule using a pituitary rongeur under arthroscopic observation. The synovium tissue was cut into small pieces and was then digested with 0.1% type I collagenase (Sigma) for 60 min at 37°C with vigorous shaking. Cells were then expanded in monolayers in the growth medium according to the described methods [25]. Dulbecco’s modified eagle medium (DMEM, Invitrogen, Carlsbad, CA), supplemented with 10% fetal bovine serum (FBS, Invitrogen), 100 U/mL penicillin, and 100 mg/mL streptomycin (1% P/S, Invitrogen), was used as growth medium. The medium was replaced every 2–3 days. The cells used in subsequent experiments were at passage 3 and were CD90^+^, CD105^+^, CD73^+^/CD45^−^ cells.

### Ad-BMP-12 infection

The adenoviral vector Ad-BMP-12 was constructed as we previously reported [26]. A recombinant adenoviral vector expressing green fluorescent protein (GFP) alone was used as a control vector (Ad-GFP). The three types of passage 3 MSCs were cultured in the growth medium to approximately 90% confluence, and Ad-BMP-12 was added according to the multiplicity of infection (MOI). The MSCs infected with Ad-GFP were used as control.

### Proliferation assay

The three types of MSCs were seeded in 96-well plates at a density of 5 × 10^3^ cells/well and cultured in the aforementioned growth medium at 37°C under 5% CO_2_ atmosphere for 1, 2, 3, 4, 5, and 6 days. Cell proliferation activity was measured using Cell Counting Kit-8 (CCK-8, Dojindo, Kumamoto, Japan), in which 10 μL of the CCK-8 assay solution was added to each well and incubated for 4 h at 37°C. The absorbance was measured using a microplate reader (Bio-Rad, Munich, Germany) at a wavelength of 450 nm.

### Chondrogenic, osteogenic and adipogenic differentiation

A pellet culture system was used for chondrogenic differentiation. Approximately 2.5 × 10^5^ MSCs were placed in a 15 mL tube and pelleted under centrifugation at 500 *g* for 10 min. The pellet was cultured in 500 μL of serum-free chondrogenic induction medium (RASMX-90041, Cyagen). The medium was replaced every 3 days for up to 21 days. For osteogenic differentiation, 3 × 10^3^/cm^2^ cells were cultured in the osteogenic induction medium (RASMX-90021, Cyagen) for 2–3 weeks according to the manufacturer’s instructions. For adipogenic differentiation, 2 × 10^4^/cm^2^ cells were cultured in the adipogenic induction medium and the maintenance medium (RASMD-90021, Cyagen), following manufacturer’s instruction. Cells in the control group were maintained only in the maintenance medium according to the same schedule.

### Flow cytometric analysis

For surface marker analysis, 1 × 10^6^ of each of the three types of MSCs were washed, incubated with fluorescein isothiocyanate (FITC)-conjugated CD105, CD73, CD45, and CD90 antibodies (Abcam, Cambridge, UK) in 1% FBS/PBS for 1 h. After three washes with 1% FBS/PBS, the cells were resuspended in 500 μL of PBS. For negative controls, FITC-conjugated nonspecific IgG fractions (Abcam) were substituted for the primary antibodies. All the above procedures were performed in the dark at 4°C. The expression profiles of CD105, CD73, CD45, and CD90 on the three types of MSCs were examined using a flow cytometer (B&D, San Jose, CA, USA) and analyzed with Cell-Quest 3.1 software (B&D).

For the assessment of Ad-BMP-12 infection efficiency, the three types of MSCs were first initially subjected to Ad-BMP-12 infection for 1 and 3 days at different MOIs. The infection efficiency was then evaluated using flow cytometer (B&D) and analyzed with Cell-Quest 3.1 software (B&D). The MSCs without Ad-BMP-12 infection were used as control.

### Quantitative RT-PCR

Total RNA was extracted from Ad-BMP-12 infected MSCs using TRIzol reagent (Invitrogen). Isolated RNA was reverse-transcribed with a commercial kit (Promega, Madison, WI, USA), and real-time RT-PCR analysis was performed using the Step-One plus System (Applied Biosystems, Foster City, CA, USA) with SYBR Green Select Master Mix (Applied Biosystems). The conditions of real-time RT-PCR were as follows: 50°C for 2 min, 95°C for 2 min, followed by 40 cycles of 95°C for 15 s and 60°C for 30 s. A dissociation stage was added at the end of the amplification procedure. There was no nonspecific amplification determined by the dissolved curve. The PCR primers are as follows: peroxisome proliferator-activated receptor γ (PPARγ): forward: 5′ TGGAGCCTAAGTTTGAGTTTGC 3′, reverse: 5′-TGACAATCTGCCTGAGGTCTG-3′; osteocalcin (OCN): forward: 5′-GCACCACCGTTTAGGGCAT-3′, reverse: 5′-AGAGAGAGGGAACAGGGAG-3′; collagen type II (Col II): forward: 5′-CACCGCTAACGTCCAGATGAC-3′, reverse: 5′-GGAAGGCGTGAGGTCTTCTGT-3′; tenomodulin (Tnmd): forward: 5′-GGGATTGACCAGAATGAGCAA-3′, reverse: 5′-GGTGCGGCGGGTCTTC-3′; tenascin C (Tnc): forward: 5′-CAGAAGCTGAACCGGAAGTTG-3′, reverse: 5′-GGCTGTTGTTGCTAGGCACT-3′; scleraxis (SCX): forward: 5′-TGGCCTCCAGCTACATTTCT-3′, reverse: 5′-TGTCACGGTCTTTGCTCAAC-3′; collagen type I (Col I): forward: 5′-TTTCCACATGCTTTATTCCAGC-3′, reverse: 5′-TCCTGGGCCTATCTGATGATCT-3′; GAPDH: forward: 5′-GCAAGTTCAACGGCACAG-3′, reverse: 5′-GCCAGTAGACTCCACGACA-3′. The expression of the above genes relative to GAPDH were determined using the 2^−ΔΔCT^ method [27].

### Enzyme-linked immunosorbent assay

The concentration of collagen type I (Col I) secreted into the culture supernatants by MSCs after Ad-BMP-12 infection was measured using a rat Col I enzyme-linked immunosorbent assay (ELISA) kit (Chondrex, Redmond, WA, USA) according to the manufacturer’s instructions. The absorbance was measured using a microplate reader (Bio-Rad) at a wavelength of 450 nm, and the results were compared with a standard curve constructed from a standard Col I solution. We confirmed that these kits did not cross-react with the medium itself, regardless of the presence or absence of FBS.

### Protein isolation and Western blotting

Protein was extracted using lysis buffer (50 mM Tris–HCl, pH 7.4, 150 mM NaCl, 1% NP- 40, and 0.1% sodium dodecyl sulfate), and the concentration was measured using the BCA protein assay kit (Pierce, Rockford, IL, USA) using bovine serum albumin as the standard. Proteins were run on SDS-PAGE gels (12%) and electro-transferred to nitro-cellulose membrane at 4°C for 2 h. The blots were probed with anti-SCX (Abcam), anti-Tnmd (Santa Cruz, Santa Cruz, CA, USA) and anti-Tnc (Santa Cruz) at 1:1,000 dilutions overnight at 4°C, followed by incubation with horseradish peroxidase-conjugated secondary antibody (Santa Cruz, 1:1,000 dilutions) at room temperature for 1 h. Proteins were detected by chemiluminescence according to the manufacturer’s recommendations (ECL, Millipore, Bedford, MA, USA). Beta-tublin (Santa Cruz) was used as an internal control.

### In vivo implantation and histological analysis

All animal experimental protocols were approved by the Animal Care and Use Committee of Peking University and conformed to the National Institutes of Health guidelines (LA 2010–066). MSCs infected with Ad-BMP-12 or Ad-GFP (4 × 10^6^) were injected subcutaneously into the right axillary region of nude mice (*n* = 8). MSCs infected with Ad-GFP were used as control. After 3 and 6 weeks, the mice were sacrificed with ether anesthesia, samples were measured with weight and area, and then were fixed in 4% paraformaldehyde (pH 7.4) for 48 h at 4°C. For standard histological evaluation, the sections were stained with hematoxylin and eosin (H&E). For immunohistochemical staining, the sections were incubated overnight at 4°C with anti-Col I, followed by a 30 min incubation with a secondary antibody conjugated with horseradish peroxidase (1:1,000, Santa Cruz) in 0.1% PBS. Col I was quantified using Image-Pro Plus 6.0 software (Media Cybernetics, Silver Spring, MD, USA).

### Statistical analysis

For statistical analysis, the statistical significance of the differences between groups was calculated using analysis of variance (ANOVA). The results from the same group were evaluated using Student’s t test. P values <0.05 were considered statistically significant. All data are presented as mean ± SD.

## Results

### Characteristics of MSCs from bone marrow, adipose tissue, and synovium

**Figure 1:**
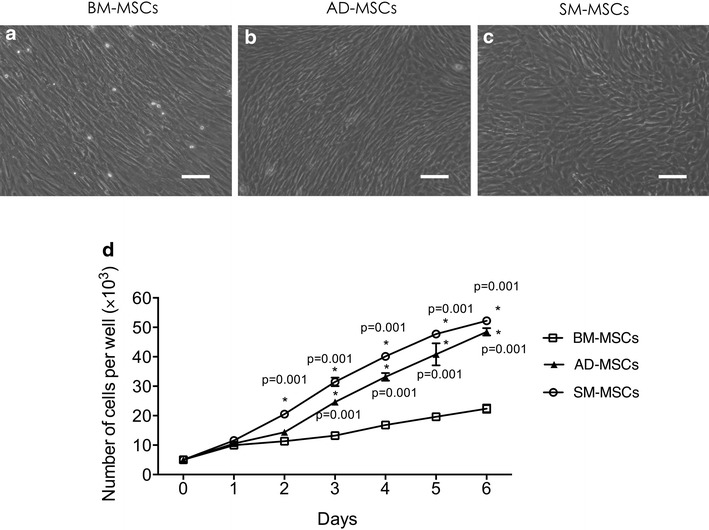
Morphology and proliferation differences among three types of MSCs. **a**–**c** BM-MSCs, AD-MSCs, and SM-MSCs were isolated from the bone marrow, adipose tissue, and synovium, respectively. Images show the MSCs at passage 3. Magnification ×10, *bar* 50 µm. **d** All three types of MSCs were seeded in a 96-well plate at 5 × 10^3^ per well and were cultured for 1, 2, 3, 4, 5, and 6 days. The cell numbers at each day were measured using CCK-8 assay. The cells on day 0 were used as control. The data were obtained from three independent experiments, each performed in triplicate. Each *bar* represents mean ± SD (*p < 0.05, vs. BM-MSCs, at the same time point, ANOVA).

**Figure 2:**
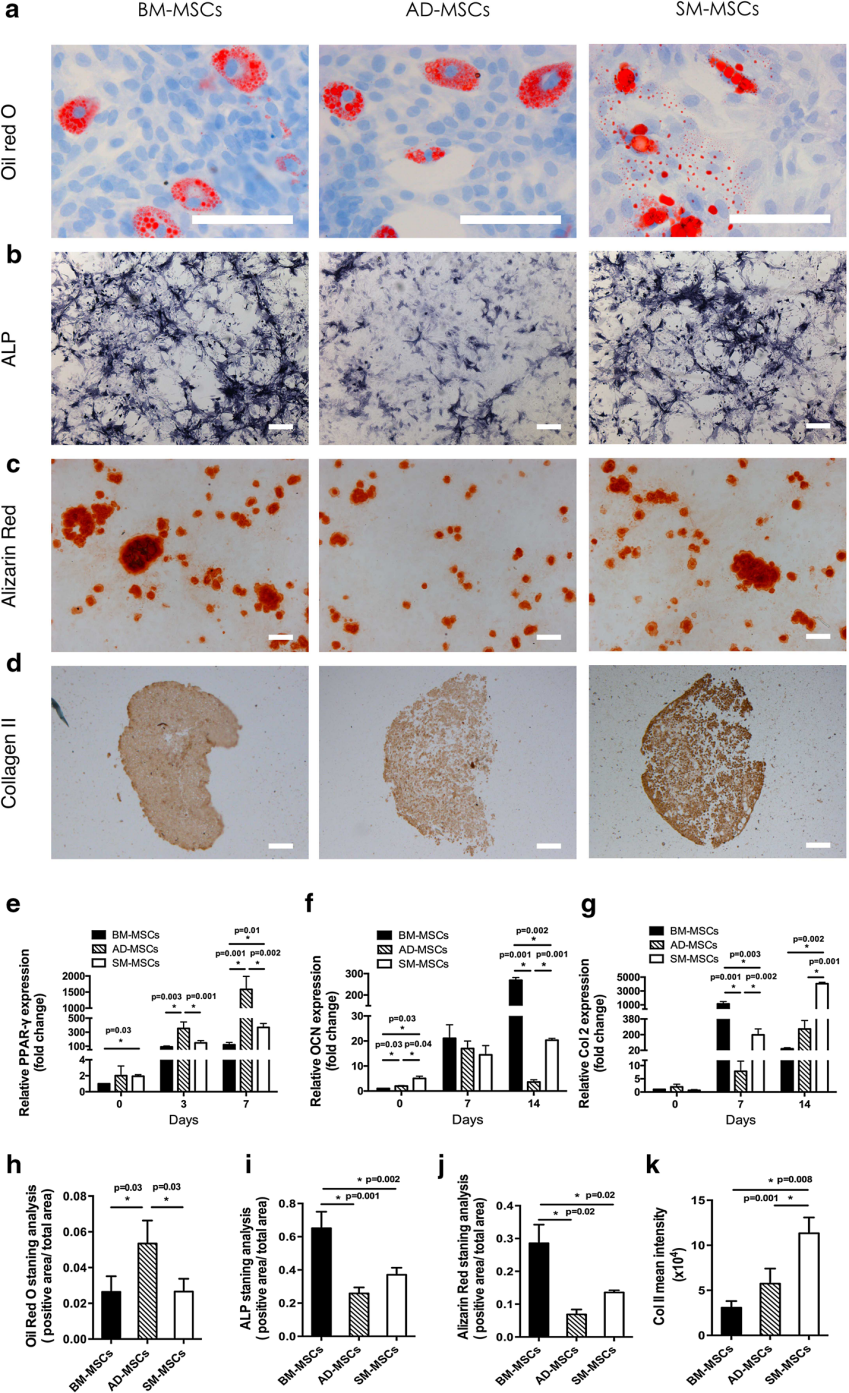
Comparative analysis of the tri-lineage differentiation capacities of the three types of MSCs. **a** All three types of MSCs were cultured in adipogenic differentiation medium for 14 days and were assessed by oil *red* O staining (used to identify adipogenic differentiation). Magnification ×10, *bar* 50 µm. **b** and **c** All three types of MSCs were cultured in osteogenic differentiation medium for 21 days and were assessed by ALP (alkaline phosphatase, a byproduct of osteoblast activity) staining (**b**) and Alizarin *red* staining (used to identify calcium deposits) (**c**). Magnification ×4, *bar* 50 µm. **d** All three types of MSCs were cultured in chondrogenic differentiation medium for 21 days and were assessed by immunohistochemical staining with anti-type II collagen antibody. Magnification ×4, *bar* 50 µm.** e** PPAR-γ (adipogenic differentiation marker) was evaluated by real-time RT–PCR at 0, 3, and 7 days post induction (mean ± SD, *n* = 3, *p < 0.05, ANOVA). **f** and **g** OCN (osteogenic differentiation marker) (**f**) and Col II (chondrogenic differentiation marker) (**g**) were evaluated by real-time RT–PCR at 0, 7, and 14 days post induction (mean ± SD, *n* = 3, *p < 0.05, ANOVA). **h**–**j** Analysis of oil *red* O staining (**h**), ALP staining (**i**), and alizarin *red* staining (**j**). The ratio of positive-stained area to the total area of cells was calculated using Image-Pro Plus 6.0 software (mean ± SD, *n* = 5, *p < 0.05, ANOVA). **k** Analysis of immunohistochemical staining of collagen type II. Expression levels were quantified by mean intensity of the images analyzed using Image-Pro Plus 6.0 software (mean ± SD, *n* = 5, *p < 0.05, ANOVA).

Isolated MSCs from the three types of tissues showed a fibroblast-like morphology (Figure 1a–c). MSCs (5 × 10^3^ cells/well in 96-well plates) were cultured for 6 days to compare the proliferation capacities of MSCs from bone marrow, adipose tissue, and synovium. Significant difference was not observed in cell numbers on day 1. However, higher proliferation capacity was observed in SM-MSCs, which was consistent with other reports [28], followed by AD-MSCs and then BM-MSCs from day 2 to day 6 (Figure 1d, p = 0.001). Under the same induction medium, SM-MSCs established the most superior chondrogenic potential (Figure 2d, g, and k; Figure 2g: p = 0.002 vs. BM-MSCs, p = 0.001 vs. AD-MSCs; Figure 2k: p = 0.008 vs. BM-MSCs, p = 0.001 vs. AD-MSCs). BM-MSCs exhibited the most superior osteogenic differentiation potential according to OCN expression (Figure 2f: p = 0.002 vs. SM-MSCs, p = 0.001 vs. AD-MSCs) and the staining results of ALP and Alizarin red (Figure 2b, c, i, j; Figure 2i: p = 0.002 vs. SM-MSCs, p = 0.001 vs. AD-MSCs; Figure 2j: p = 0.02 vs. SM-MSCs, p = 0.02 vs. AD-MSCs).

**Figure 3:**
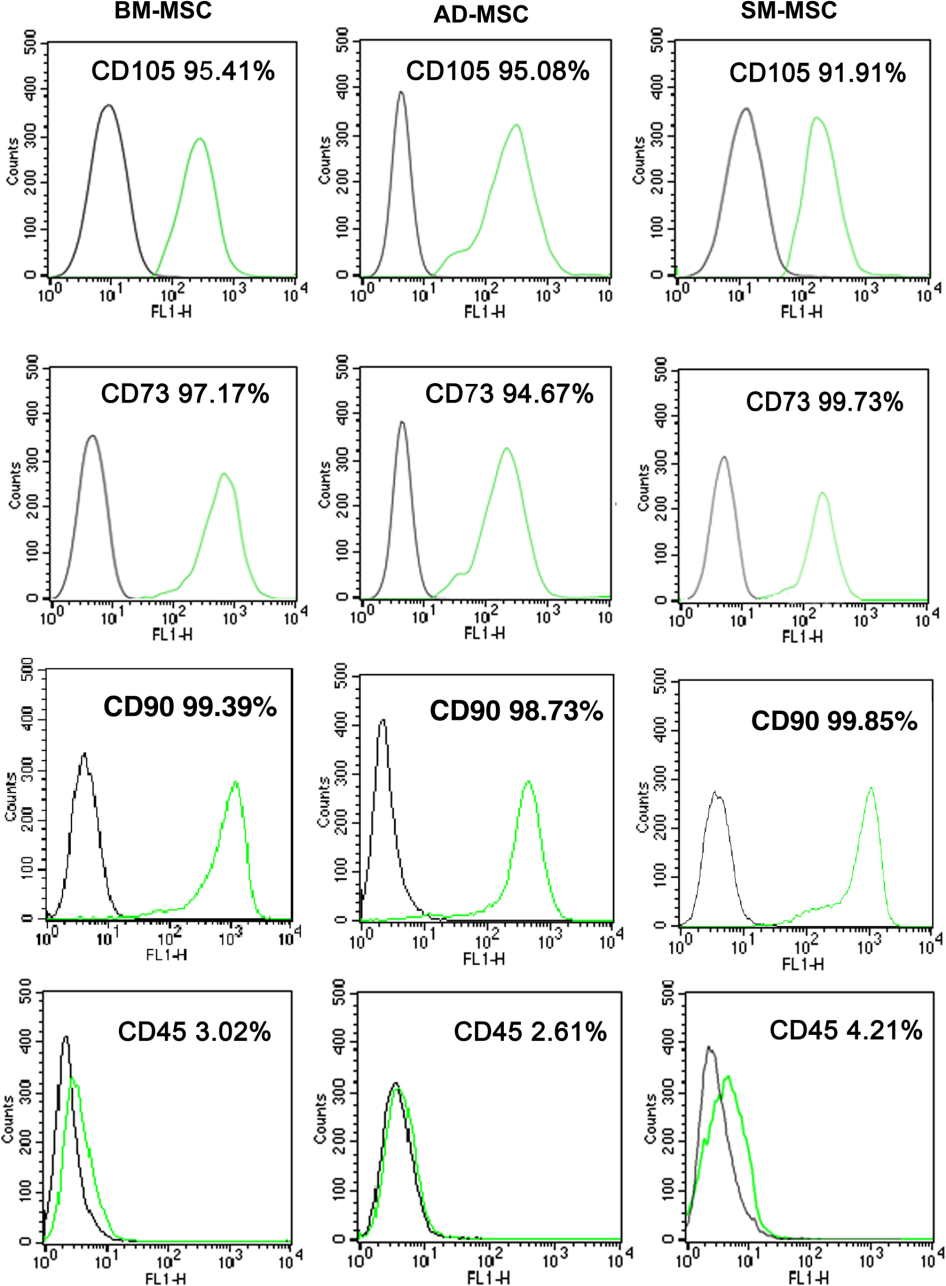
Immunophenotypic profiles of MSCs derived from three different rat tissues. Three types of MSCs at passage 3 (1 × 10^6^) were incubated with FITC-conjugated CD105, CD73, CD90, and CD45 antibodies (Abcam, Cambridge, UK) in 1% FBS/PBS for 1 h. Cells were washed three times with 1% FBS/PBS and then resuspended in 500 μL of PBS. For negative controls, FITC-conjugated nonspecific IgG fractions (Abcam) were substituted for the primary antibodies.

All three types of MSCs ubiquitously expressed CD105, CD73, and CD90 (CD105: 95.41% in BM-MSCs, 95.08% in AD-MSCs, and 91.91% in SM-MSCs; CD73: 97.17% in BM-MSCs, 94.67% in AD-MSCs, and 99.73% in SM-MSCs; CD90: 99.39% in BM-MSCs, 99.85% in AD-MSCs, and 98.73% in SM-MSCs) (Figure 3). However, all three types of MSCs did not express the hematopoietic marker CD45 (Figure 3).

### In vitro tenogenic differentiation capacities of MSCs from bone marrow, adipose tissue, and synovium after Ad-BMP-12 infection

**Figure 4:**
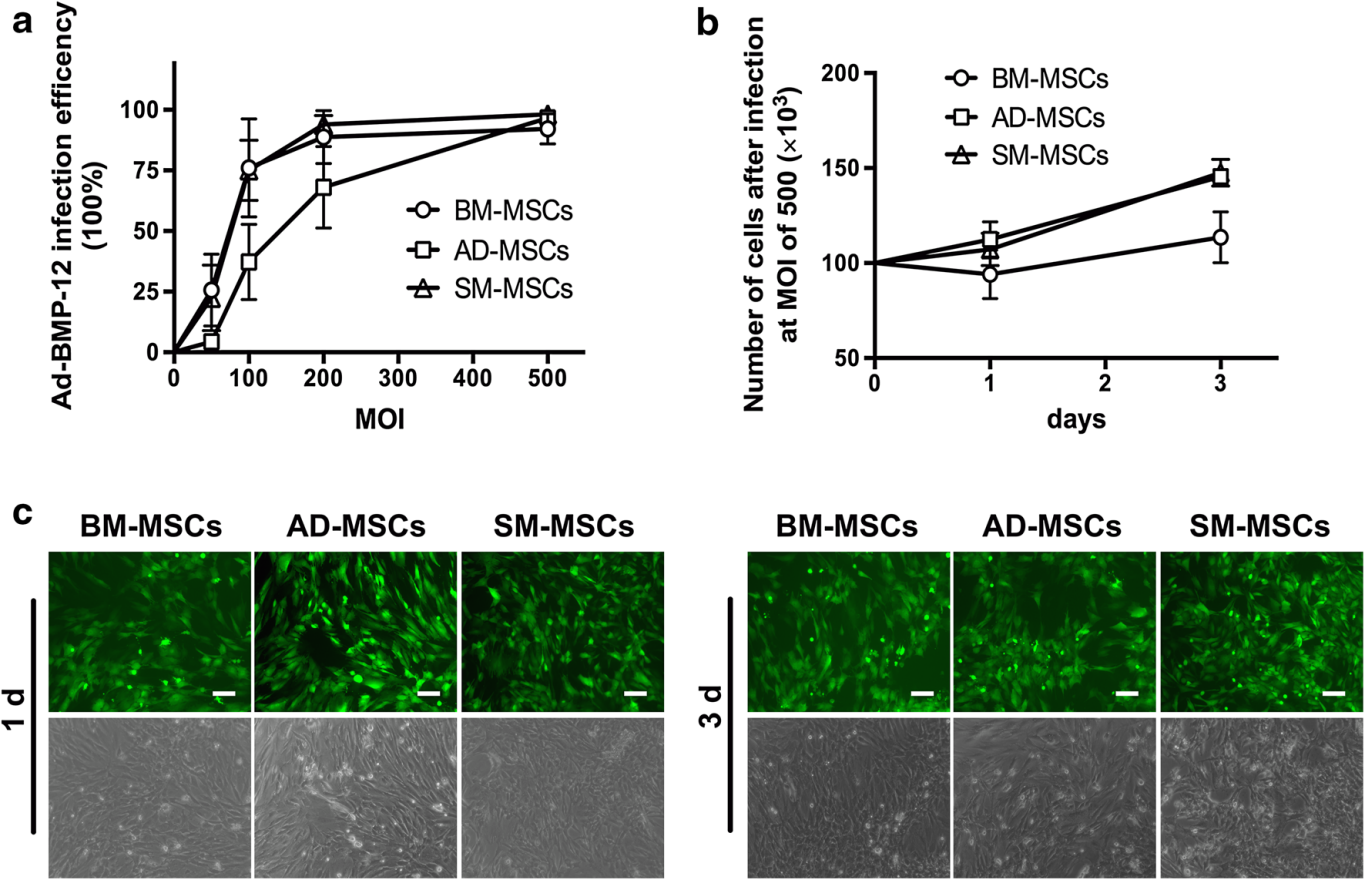
Influence of recombinant Ad-BMP-12 virus on cell viability. **a** All three types of MSCs were infected with Ad-BMP-12 at MOIs of 50, 100, 200, and 500. Untreated MSCs were used as controls. Flow cytometry was used to assess infection efficiency. The data were obtained from three independent experiments, each performed in triplicate. Each *bar* represents mean ± SD (*p < 0.05, vs. control, t test). **b** All three types of MSCs were seeded in a 24-well plate at 1 × 10^5^ per well and infected with Ad-BMP-12 at MOI of 500. Cell numbers were measured using CCK-8 assay on days 1 and 3. The data were obtained from three independent experiments, each performed in triplicate. Each *bar* represents mean ± SD (*p < 0.05, vs. control, t test). **c** Images were acquired using a fluorescence microscope to show infection efficiency and cell viability on days 1 and 3. Cells with *green* fluorescence were the infected MSCs. Magnification ×10, *bar* 50 µm.

The tenogenic differentiation capacities of the three types of MSCs in the presence of BMP-12 were compared by constructing recombinant adenovirus Ad-BMP-12 as we previously reported [26]. Infection efficiency assay was used to establish a suitable infection condition. Four MOIs (0, 50, 100, and 500) were set up, and MOI of 500 was determined as the consistent infection efficiency among the three types of infected MSCs (~100%) (Figure 4a). Moreover, increased cell numbers were still observed in all MSCs from day 1 to day 3 (Figure 4b).

**Figure 5:**
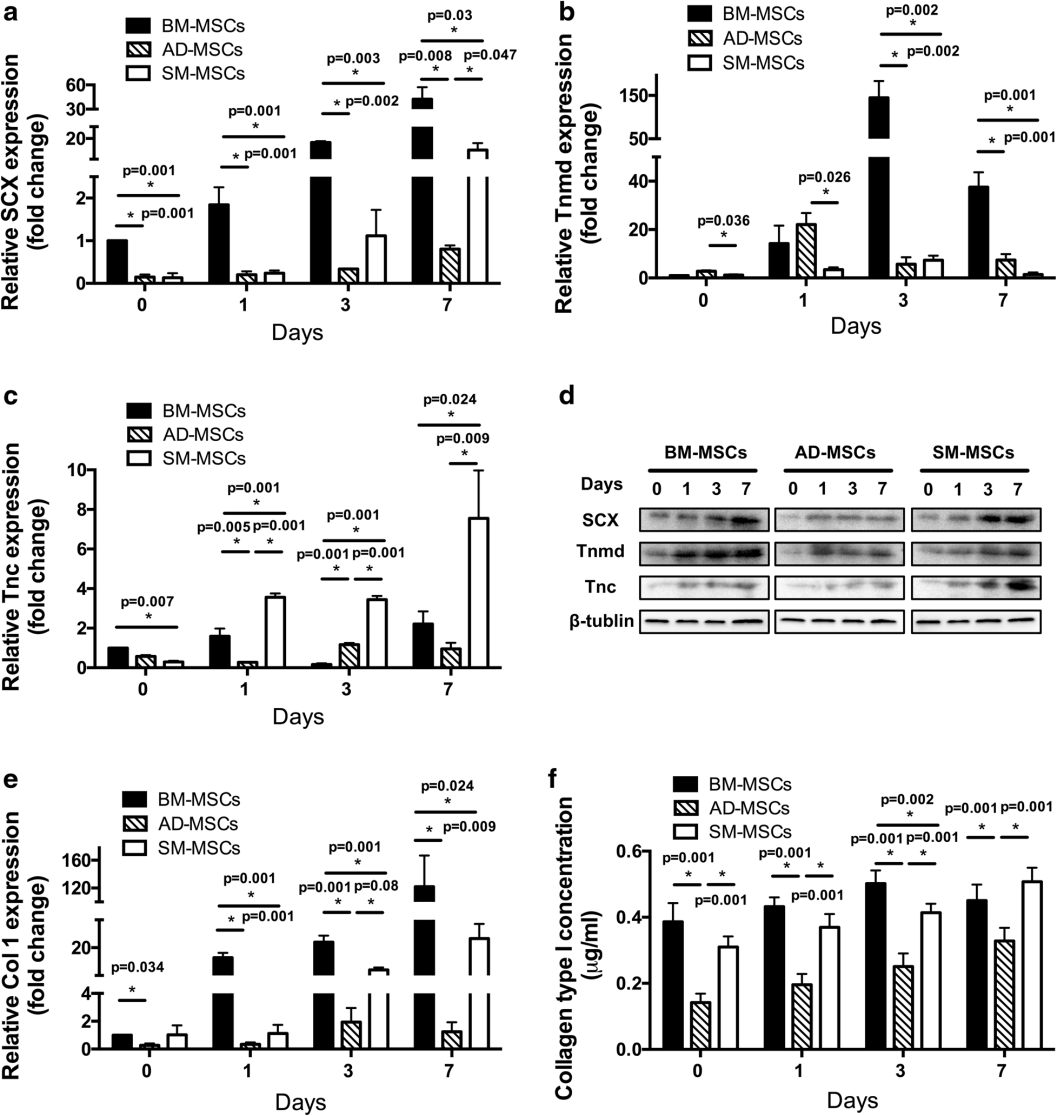
Comparative analysis of the tenogenic differentiation capacities of three types of MSCs. **a**–**c** MSCs were cultured with Ad-BMP-12 for 1, 3, and 7 days. The expression levels of the specific tenogenic genes Scx (**a**), Tnmd (**b**), and Tnc (**c**) were evaluated by real-time RT–PCR at 0, 1, 3, and 7 days after infection (each *bar* represents mean ± SD from three independent experiments; *p < 0.05, ANOVA). The BM-MSCs infected with Ad-GFP on day 0 were used as control. **d** Western blot was performed with total proteins from all three types of MSCs at 0, 1, 3, and 7 days after infection to detect the levels of Scx, Tnmd, and Tnc. β-Tublin was used as control.** e** Col I expression was evaluated by real-time RT–PCR at 0, 1, 3, and 7 days after induction (each *bar* represents mean ± SD from three independent experiments; *p < 0.05). The BM-MSCs infected with Ad-GFP on day 0 were used as control. **f** Col I concentration in the cell lysate was quantified at 0, 1, 3, and 7 days post Ad-BMP-12 infection by using a Col I ELISA kit (each *bar* represents mean ± SD from three independent experiments; *p < 0.05, ANOVA).

The effect of Ad-BMP-12 infection on the differentiation capacities of the three types of MSCs was also assessed. Tenogenic differentiation was evaluated based on the expression of tenocyte-lineage marker genes at both mRNA and protein levels. Scleraxis (SCX), a key transcription factor during tenogenic differentiation process; Tnmd and tenascin-c (Tnc), the tenogenic differentiation markers; and Col I, a major extracellular matrix protein were used to assess tenogenic differentiation. The expression levels of the aforementioned tenogenic genes were assessed at 1, 3, and 7 days after induction by real-time reverse transcription polymerase chain reaction (RT–PCR). Upregulated mRNA expression levels of all four tenogenic genes were observed in induced MSCs but with different patterns. BM-MSCs exhibited the highest expression levels of SCX (Figure 5a: 1, 3, and 7 days; p value is shown in the figure), Tnmd (Figure 5b: 3 and 7 days; p value is shown in the figure), and Col I (Figure 5e: 1, 3, and 7 days; p value is shown in the figure) among the three MSCs. SM-MSCs showed the highest Tnc expression level at 7 days. The basal expression levels of SCX, Tnmd, Tnc, and Col I (day 0) were significantly different across the three types of MSCs. BM-MSCs exhibited the highest basal level of SCX (Figure 5a, p = 0.001 vs. SM-MSCs, p = 0.001 vs. AD-MSCs), Tnc (Figure 5c, p = 0.007 vs. SM-MSCs), and Col I (Figure 5e, p = 0.034 vs. AD-MSCs). By contrast, AD-MSCs had the highest basal level of Tnmd (Figure 5b, p = 0.036 vs. SM-MSCs).

Upregulated protein expressions of all four tenogenic markers were observed in the induced MSCs with time duration. The highest expression levels of SCX, Tnmd, and Col I were observed in BM-MSCs, whereas the highest expression level of Tnc was observed in SM-MSCs (Figure 5d, p = 0.024 vs. BM-MSCs, p = 0.009 vs. AD-MSCs).

All these data suggest the most superior tenogenic differentiation capacity of BM-MSCs, followed by SM-MSCs and then AD-MSCs in vitro.

### Comparative analysis of in vivo tendon-like tissue formation in the three types of MSCs

**Figure 6:**
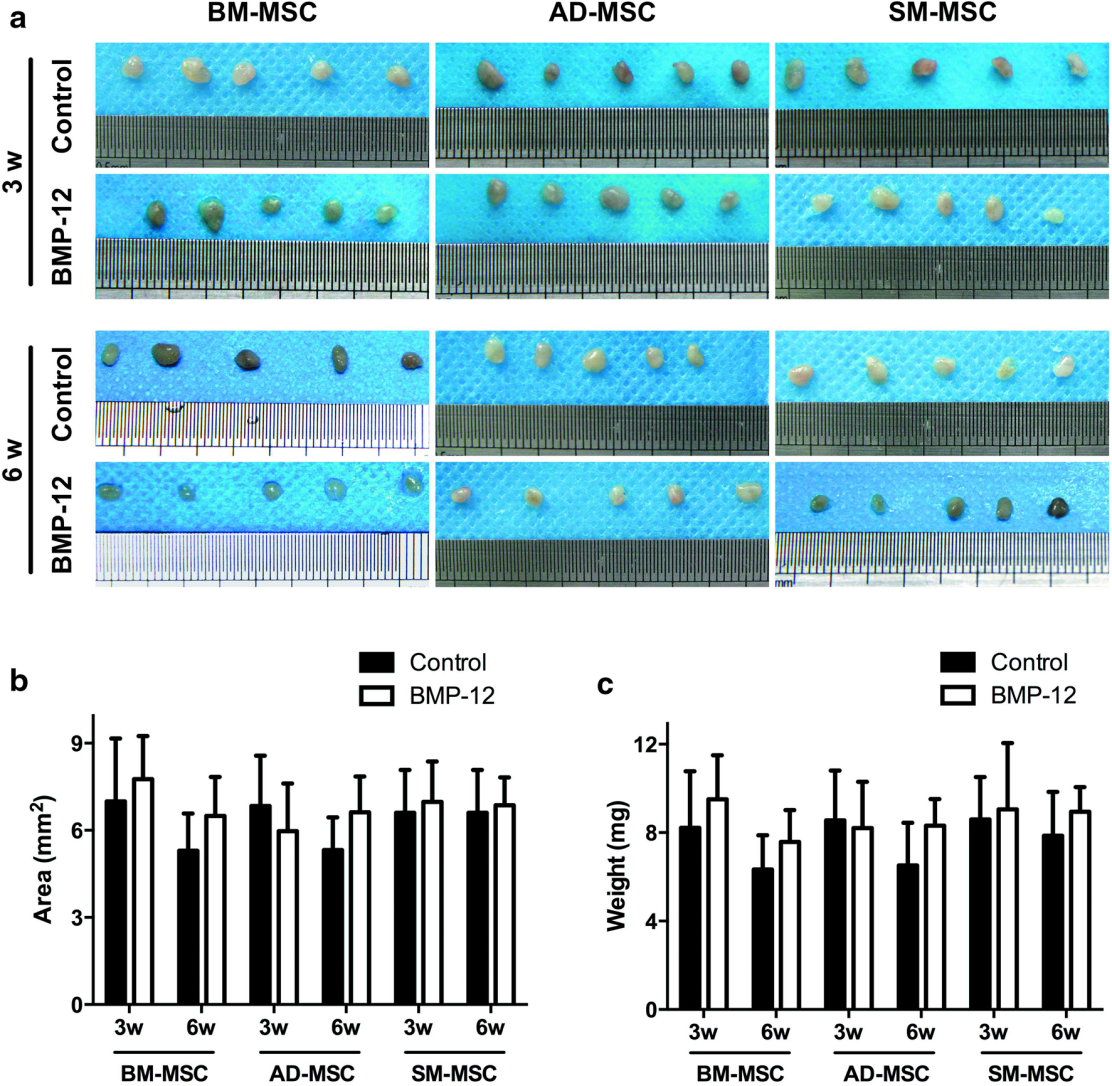
Properties of the implanted Ad-BMP-12 infected cell masses. **a** MSCs were initially treated with Ad-BMP-12 for 1 day and were then subcutaneously injected into the nude mice for 3 or 6 weeks. The MSCs treated with Ad-GFP were used as control. Images show the morphologies of the cell masses after 3 and 6 weeks from injection. **b** and **c** Comparison of the three types of MSCs in terms of the area (**b**) and weight (**c**) of the cell masses. (Each *bar* represents mean ± SD; *P < 0.05, statistical method: t test: 3w/6w BMP-12 group vs. 3w/6w control group, respectively; ANOVA: 3w/6w BMP-12 group in BM-MSC vs. 3w/6w BMP-12 group in AD-MSC vs. 3w/6w BMP-12 group in SM-MSC, respectively).

**Figure 7:**
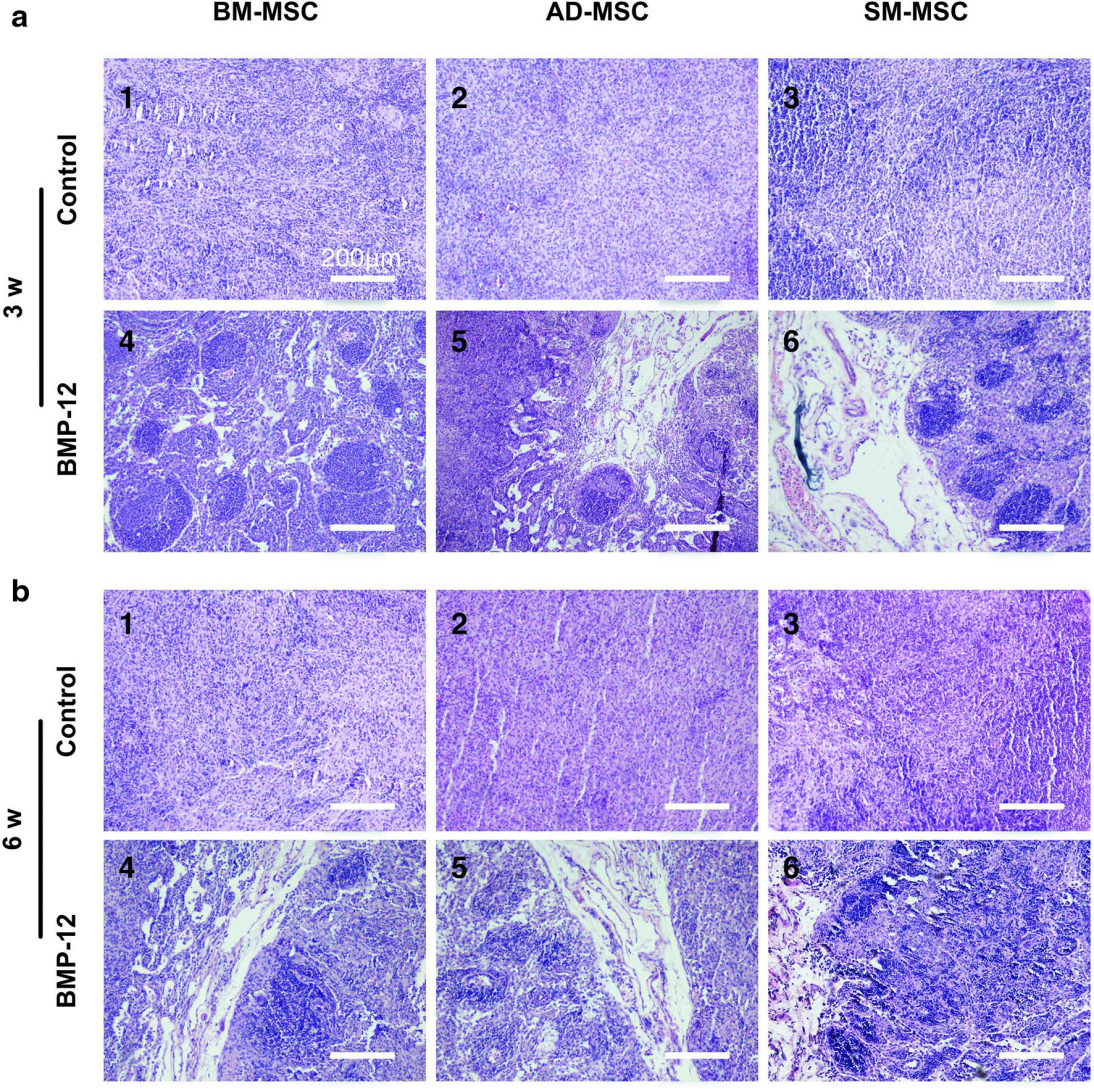
H&E staining of the implanted cell masses. The specimens were fixed and embedded in paraffin. Sections of 5 µm cut through the center of the cell masses were stained with hematoxylin/eosin (H&E). Images show the staining at 3 weeks (**a**) and 6 weeks (**b**). Magnification ×10, *bar* 200 µm.

**Figure 8:**
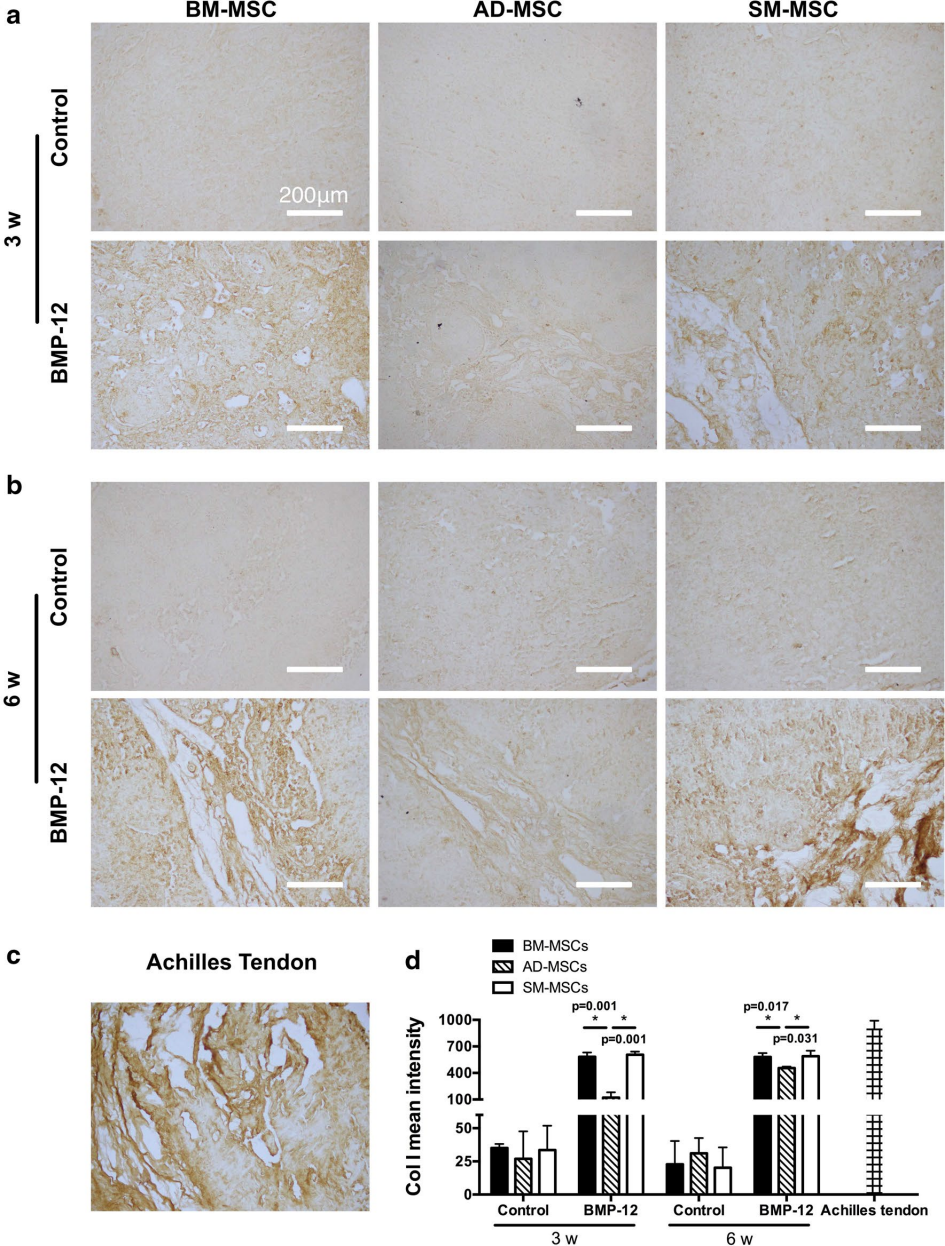
Immunohistochemical analysis of implanted MSCs. **a** and **b** The specimens were fixed and embedded in paraffin. Sections of 5 µm cut through the center of the cell masses were stained with anti-Col I antibody. Images show the staining at 3 weeks (**a**) and 6 weeks (**b**). Magnification ×10, *bar* 200 µm. **c** Immunohistochemical staining of Col I of rat Achilles tendon was used as positive control. **d** Col I expression was quantified by mean intensity of the images analyzed using Image-Pro Plus 6.0 software. Achilles tendon was used as positive control (each *bar* represents mean ± SD, *n* = 5; *p < 0.05, ANOVA).

Different capacities of tenogenic differentiation among the three types of MSCs are demonstrated by the aforementioned results in vitro. These differences were also examined in vivo. All three types of MSCs were initially infected with Ad-BMP-12 for 24 h and then were injected subcutaneously into the right axillary region of the nude mice. After 3 and 6 weeks, these injected cell masses were removed for further analysis. As shown in Figure 6a, these cells were aggregated together, and most of them presented ellipse-shaped tissues. Significant difference between 3- and 6-week samples was not detected at the area and weight of the three types of MSCs (Figure 6b, c). Tenogenic differentiation was evaluated based on the histological findings. No difference was observed in the macroscopic morphologies of the cells in all groups; however, they exhibited different cell arrangements, as shown in the hematoxylin/eosin images. Furthermore, regardless of the type of MSCs, the organization of the cells infected with Ad-BMP-12 was different from that of the cells infected with Ad-GFP (Figure 7). The organization and arrangement of the control MSCs were poorly organized, compact, and uniform (Figure 7a1–3, b1–3). By contrast, the specimens of the MSCs infected with Ad-BMP-12 exhibited a special structure. Some of the cells were aggregated together and formed numerous small cell masses, whereas some parts of the tissues emerged in the form of fiber-like matrix, especially in 6-week specimens (Figure 7a4–6, b4–6). Immunohistochemical staining for Col I was used to assess matrix production after Ad-BMP-12 infection. Rat Achilles tendon was employed as positive control; the staining intensity was quantified by mean intensity (Figure 8). Compared with AD-MSCs, BM-MSCs and SM-MSCs revealed more intense staining for Col I in both 3- and 6-week specimens (Figure 8a, b, d; 3w: BM-MSC vs. AD-MSCs: p = 0.001, SM-MSC vs. AD-MSCs: p = 0.001; 6w: BM-MSC vs. AD-MSCs: p = 0.017, SM-MSC vs. AD-MSCs: p = 0.031). Difference was not observed between BM-MSCs and SM-MSCs at both 3 and 6 weeks (Figure 8a, b, d).

## Discussion

Tendons are poorly vascularized tissues and thus have difficulty regenerating. Tendon repair is slow and inefficient after injury. Tenocytes are highly differentiated cells and have low cell density. Tenocytes are embedded in 3D extensive extracellular matrix and exhibit low proliferation activities. Cell-based regenerative medicine appears to have great prospect for clinical applications in tendon repair. Screening for an appropriate cell source for tendon repair is crucial. The criteria for an ideal cell source have been proposed [29]. First, the cells should have rapid proliferation capacity because healthy cells should be adequate for implantation in vitro. Second, cells should be easy to harvest. The cells should be easily isolated without substantial secondary trauma to the harvest site. Finally and most importantly, cells should have effective repair capacity. A crucial problem is whether the harvested cells can easily and effectively repair the injured site.

MSCs are potential cell source with considerable clinical applications [30, 31]. MSCs are easy to isolate and possess properties, such as self-renewal, low immunogenicity, and multilineage differentiation capabilities [32–34]. MSCs can be virtually isolated from many tissues, such as bone marrow, adipose, muscle, and synovium. BM-MSCs have received considerable attention from researchers for their remarkable plasticity and significant differentiation potential. In recent years, other MSC sources have been identified. AD-MSCs isolated from adipose tissues can be easily isolated by minimally invasive techniques. Similar to BM-MSCs, AD-MSCs are considered a promising therapeutic cell source because of their multilineage differentiation potential [35, 36]. SM-MSCs have great differentiation potential toward adipogenesis, osteogenesis, chondrogenesis, and myocyte, suggesting high multipotency [37, 17]. SM-MSCs are considered a new source of MSCs for regenerative medicine for the musculoskeletal system [25]. MSCs from different tissues differ in proliferation, isolation, and differentiation capacities. For example, SM-MSCs and AD-MSCs are superior in adipogenesis, whereas BM-MSCs, SM-MSCs, and periosteum-derived MSCs are superior in osteogenesis [17]. Our previous studies elucidated the mechanisms of osteogenic differentiation of BM-MSCs and AD-MSCs and presented differences between these mechanisms [18]. Little is still known about the differences in tenogenic differentiation capacities. Therefore, identifying and characterizing the appropriate cell source for tendon repair are crucial.

In this study, we compared the proliferation capacities, trilineage differentiation capacities, and tenogenic differentiation potentials of MSCs. The MSCs were derived from rat bone marrow, adipose tissue, and synovium in the presence of BMP-12. SM-MSCs exhibited the most superior proliferation capacity, followed by AD-MSCs and BM-MSCs, which was consistent with the findings of previous reports [17, 38]. Furthermore, the tenogenic differentiation capacities among these MSCs were significantly different, with BM-MSCs exhibiting the most superior tenogenic differentiation capacity, followed by SM-MSCs and AD-MSCs, in the presence of BMP-12 both in vivo and in vitro.

Results showed that BM-MSCs exhibited the most superior tenogenic differentiation potential but the most inferior proliferation capacity. Painful biopsy procedure is another limitation in using BM-MSCs [39, 40]. AD-MSCs are considered a promising cell source because the adipose tissue is ubiquitous and easily obtained, with less donor site morbidity [41]. However, in this study, among these three MSCs, AD-MSCs exhibited the most inferior tenogenic differentiation capacity. SM-MSCs demonstrated superiority in multipotency and proliferation potential compared with the other MSCs [17]. SM-MSCs also showed good tenogenic differentiation potential both in vivo and in vitro. Unfortunately, SM-MSCs are always harvested via arthroscopy, which is an invasive procedure.

In summary, BM-MSCs and SM-MSCs showed the most superior tenogenic differentiation and cell proliferation capacities, respectively. AD-MSCs showed the most inferior proliferation and tenogenic differentiation capacities.

The results of this study provide useful information on selecting the optimum MSCs to improve tendon repair. Further study on MSCs of other species, especially human MSCs, should be performed and verified.

## Conclusion

BM-MSCs exhibited the most superior tenogenic differentiation capacity, followed by SM-MSCs. By contrast, AD-MSCs demonstrated the most inferior capacity among the three types of MSCs in the presence of BMP-12 both in vivo and in vitro. This study provides useful information on selecting the optimum MSCs to improve tendon repair.

## Abbreviations

MSCs: mesenchymal stem cells
BMP-12: bone morphogenic protein 12
BM-MSCs: bone marrow-derived MSCs
AD-MSCs: adipose-derived MSCs
SM-MSCs: synovial membrane-derived MSCs
BMPs: bone morphogenetic proteins
TGF-β: transforming growth factor-beta
FGF: fibroblast growth factor
GDF-7: growth and differentiation factor 7
Ad-BMP-12: BMP-12 recombinant adenovirus
SCX: scleraxis
Col I: collagen type I
MOI: multiplicity of infection
PPARγ: peroxisome proliferator-activated receptor γ
OCN: osteocalcin
Col II: collagen type II
Tnmd: tenomodulin
Tnc: tenascin C
